# Filling ability of ready-to-use or powder-liquid calcium silicate-based sealers after ultrasonic agitation

**DOI:** 10.1590/0103-6440202405802

**Published:** 2024-07-22

**Authors:** Mário Tanomaru-Filho, Maíra Bonassi Lucchesi, Airton Oliveira Santos-Junior, Karina Ines Medina Carita Tavares, Jáder Camilo Pinto, Juliane Maria Guerreiro-Tanomaru

**Affiliations:** 1 Department of Restorative Dentistry, School of Dentistry, UNESP - Universidade Estadual Paulista, Araraquara, SP, Brazil; 2 Departament of Dentistry - Centro Universitário Presidente Antônio Carlos - UNIPAC, Barbacena, MG, Brazil and Department of Dentistry - Centro Universitário Presidente Tancredo de Almeida Neves - UNIPTAN, São João del Rei, MG, Brazil

**Keywords:** Endodontics, physical properties, root canal filling, ultrasonic, x-ray microtomography

## Abstract

This study evaluated the effect of ultrasonic agitation on the filling capacity of ready-to-use calcium silicate-based sealer Bio-C Sealer (BCS, Angelus, Paraná, Brazil) or powder-liquid BioRoot RCS (BR, Septodont, Saint-Maur-des-Fossés, France) using curved artificial canals by micro-computed tomography (micro-CT). Additionally, flow (mm) and flow area (mm^2^) were evaluated for both materials. Acrylic resin main canal (60° curvature and 5 mm radius, with 3 lateral canals in the cervical, middle, and apical thirds) were prepared up to size 40/.05 (Prodesign Logic, Brazil). The agitation method was used with ultrasonic tip (US, Irrisonic, Helse, Brazil): BCS, BCS/US, BR, and BR/US. All specimens were filled using the single-cone technique. The samples were scanned by micro-CT (8,74 µm) after obturation. The percentage of filling material and voids were calculated. Flow was evaluated based on ISO 6876/2012 standards (mm) and area (mm^2^). The data were statistically analyzed using ANOVA and Tukey tests (α = 0.05). BR/US showed lower percentage of filling material in the lateral canals than and, BCS/US (p<0.05). BR/US resulted in a higher percentage of voids than BR in the lateral apical third (p<0.05). BCS showed higher flow than BR (p<0.05). BCS and BR presented proper filling capacity in the simulated curved canals regardless of the use of ultrasonic agitation. However, BR/US showed more voids in the apical third. BCS demonstrates higher filling ability.

## Resumo

Este estudo avaliou o efeito da agitação ultrassônica na capacidade de preenchimento de cimento pronto para uso à base de silicato de cálcio Bio-C Sealer (BCS, Angelus, Paraná, Brasil) ou pó-líquido BioRoot RCS (BR, Septodont, Saint-Maur -des-Fossés, França) utilizando canais artificiais curvos por microtomografia computadorizada (micro-CT). Adicionalmente, escoamento (mm) e área de escoamento (mm2) foram avaliados para ambos materiais. Please, replace the sentence highlighted in yellow to: O canal principal de resina acrílica (curvatura de 60° e raio de 5 mm, com 3 canais laterais nos terços cervical, médio e apical) foi preparado até o tamanho 40/.05 (Prodesign Logic, Brasil). O método de agitação foi utilizado com ponta ultrassônica (US, Irrisonic, Helse, Brasil): BCS, BCS/US, BR e BR/US. Todos os espécimes foram obturados usando a técnica de cone único. As amostras foram escaneadas por micro-CT (8,74 µm) após obturação. A porcentagem de material obturador e vazios foram calculados. O escoamento foi avaliado com base nas normas ISO 6876/2012 (mm) e área (mm2). Os dados foram analisados estatisticamente pelos testes ANOVA e Tukey (α = 0,05BR/US apresentou menor percentual de material obturador nos canais laterais que BCS e BCS/US (p<0,05). BR/US resultou em maior porcentagem de vazios que o BR no canal lateral do terço apical (p<0,05). BCS apresentou maior escoamento que BR (p<0,05)." BCS e BR apresentaram capacidade de preenchimento adequada nos canais curvos simulados independente do uso de agitação ultrassônica. No entanto, BR/US apresentou mais vazios no terço apical. BCS demonstra maior capacidade de preenchimento.

## Introduction

Proper filling of the root canal system is a key factor in achieving higher rates of endodontic treatment success ^1^. Voids in the filling provide a greater chance for reinfection ^2^ ^,^ ^3^ ^,^ ^4^. Therefore, different obturation techniques have been proposed to optimize root canal filling ^3^. The use of an endodontic sealer is essential to fill the spaces between gutta-percha and root canal walls, as well as areas of anatomical complexities, such as curvatures and lateral canals ^1^ ^,^ ^2^ ^,^ ^3^. Endodontic sealers must have adequate flow, aiming to fill irregularities in the root canal system ^1^. According to the ISO 6876/2012 standards ^5^, more than 17 mm flow is required for the sealer. In addition, the area occupied by material, expressed in mm^2^, can be used as a complementary flow analysis ^6^.

Calcium silicate-based sealers are commercially available in ready-to-use or powder-liquid forms ^7^. The ready-to-use presentation demands the moisture of the root canals for setting, and the powder-liquid sealer initiates the hydration reaction in the presence of water during the manipulation of the material previously to insertion in the root canal ^8^. Bio-C Sealer (Angelus, Londrina, Paraná, Brazil) is a premixed, ready-to-use endodontic sealer based on calcium silicates. Adequate physicochemical and biological properties have been related to this material ^9^ ^,^ ^10^, including filling capacity in flattened root canals ^2^ ^,^ ^3^, as well as in the apical third of curved canals of lower molars ^11^. BioRoot RCS (Septodont, St. Maur-des-Fossés, França) is a powder-liquid sealer, and the liquid is fabricated based on water with calcium chloride and polycarboxylate ^12^. Proper biological and physicochemical properties are described for BioRoot RCS ^13^ ^,^ ^14^. However, a higher percentage of voids than AH Plus ^15^ and GuttaFlow BioSeal ^16^ have been reported for BioRoot RCS.

Bioceramic sealers are often associated with the single-cone filling technique ^2^ ^,^ ^3^. However, this technique requires sealer with adequate flow ^3^ for proper filling of root canals with anatomical complexities, such as curvatures and lateral canals ^11^. Ultrasonic agitation of endodontic sealer before insertion of the gutta-percha cone has been proposed as a resource to optimize the filling of root canals with anatomical complexities ^17^ ^,^ ^18^. On the other hand, it has also been reported that ultrasonic agitation does not improve the penetration of bioceramic materials into intratubular dentin ^19^ ^,^ ^20^. To date, there is no data in the literature on the effect of ultrasonic agitation on the filling capacity of ready-to-use calcium silicate-based sealer or in powder-liquid presentation.

Therefore, the aim of this study was to evaluate using micro-computed tomography (micro-CT) an influence of ultrasonic agitation on the filling capacity of ready-to-use Bio-C Sealer or powder-liquid BioRoot RCS in simulated curved canals, besides the flow of these materials using conventional ISO methodology and complementary analysis. The null hypotheses were that ultrasonic agitation would not influence the filling capacity for the different sealers and that there would be no difference in the flow for both materials.

## Material and Methods

### Sample size calculation

The sample size for this study was calculated by G* Power software (3.1.7 for Windows, Heinrich Heine, Universität Dusseldorf, Germany). One-way ANOVA test was used with an Alpha-type error of .05 and a Beta power of .99. The effect size of 1.27 was determined based on a previous study that used a similar methodology ^21^. A total of 5 specimens per group was indicated as the ideal size required, thus, an n=6 was used to compensate for possible losses during methodology implementation. 

### Preparation of the curved artificial canals

**Figure 1:**
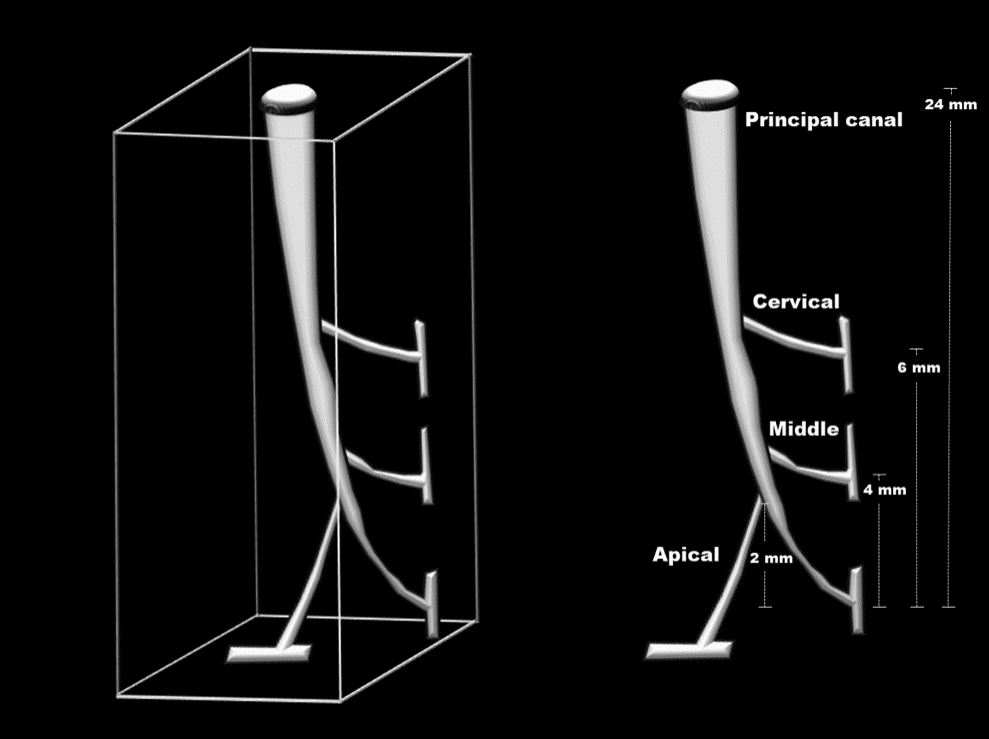
Representative image of the acrylic resin model with standard size of the simulated curved principal canal and lateral canals in the cervical, middle, and apical third.

Acrylic resin models with a curved main canal and three simulated lateral canals in the cervical, middle, and apical third (n=24) were used (IM do Brasil Ltda, São Paulo, SP, Brazil). The curved main canal had a standard size of 24 mm, 60° angle of curvature, and 5 mm radius and the center of the curvature was 5 mm from the end of the canal. The simulated lateral canals were positioned 2, 4, and 6 mm from the apical foramen, representing the apical, middle, and cervical simulated lateral canals, respectively (Figure 1). The working length (WL) was determined using a #10 K-file (Dentsply Maillefer, Ballaigues, Switzerland) 1 mm short of the simulated apical foramen. All curved main canals were prepared with a ProDesign Logic rotary system (Easy Equipamentos Odontológicos, Belo Horizonte, Minas Gerais, Brazil) operated by an electric motor (VDW Silver, VDW GmbH, Munich, Germany). The 25/.01 instrument was used at a speed of 350 rpm and torque of 1 N.cm. Then, the instruments 25/.05, 35/.05, and 40/.05 were used at a speed of 600 rpm and torque of 3 N.cm. All instruments were applied with in-and-out movements up to the WL. The simulated curved canals were irrigated with 2.5 mL of distilled water after each instrument, using a 5 mL syringe and NaviTip 27-G needle (Ultradent Products, South Jordan, UT) 2 mm short of the WL ^22^. Subsequently, all canals were carefully dried using 2 tips of #40 absorbent paper (Dentsply Maillefer) in order not to cause excessive drying, according to the protocol described by Pinto et al. ^11^. 

### Obturation of the curved artificial canals

After preparation, the curved artificial canals were divided into 4 experimental groups (n=6) for obturation using the single-cone technique and one of the sealers in the different experimental conditions. All information about the sealers, composition, manufacturers, proportions, and experimental groups is shown in Box 1. For the canals filled with Bio-C Sealer, it was injected into the simulated canals approximately 4 mm short of the WL, using syringe and plastic needles provided by the manufacturer. BioRoot RCS was manipulated according to the manufacturer´s specifications and inserted into the canal using a #40 K-file (Dentsply Maillefer) pre-curved in the WL, and lentulo spiral #40 (Dentsply Maillefer) operated clockwise in low-speed motor (Micromotor N270) and contra-angle (Dabi-Atlante, Ribeirão Preto, São Paulo, Brazil) 2 mm short of the WL. For the groups with agitation, this was performed using an Irrisonic ultrasonic tip (Helse Ultrasonic, Santa Rosa de Viterbo, São Paulo, SP, Brazil). The tip was activated for 40 seconds, 20 seconds in the buccal-lingual direction, and 20 seconds in the mesio-distal direction of the simulated curved canals 2 mm short of the WL after insertion of Bio-C Sealer or BioRoot RCS. A Newtron® Booster ultrasonic device (Acteon, North America, New Jersey, USA) was used at a frequency of 50 Hz and power of 10% to activate the Irrisonic tip, following the manufacturer's recommendations. After agitation, gutta-percha master points size 40 taper 0.05 (Tanari industry Ltda., São Paulo, Brazil) that were previously selected based on tip diameter and taper using Profilometer device (Profile Projector Nikon model 6C-2) were inserted into each simulated canal up to WL. For all experimental groups, gutta-percha excesses were cut at the cervical level with a heat plugger (Paiva #2; Golgran, São Caetano do Sul, São Paulo, Brazil). All the specimens were stored in an oven at 37° C and 95% humidity for 72 hours for the final setting of the sealers. 

**Box 1:**
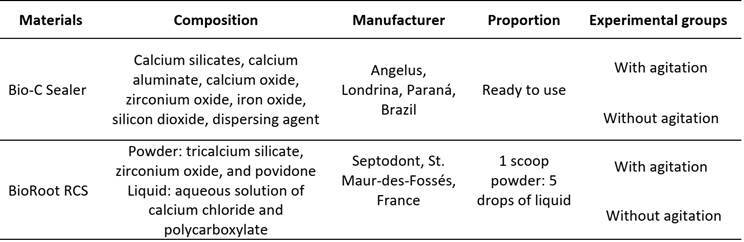
Materials used, their composition, manufacturer, proportion, and experimental groups

### Micro-CT Analysis

**Figure 2:**
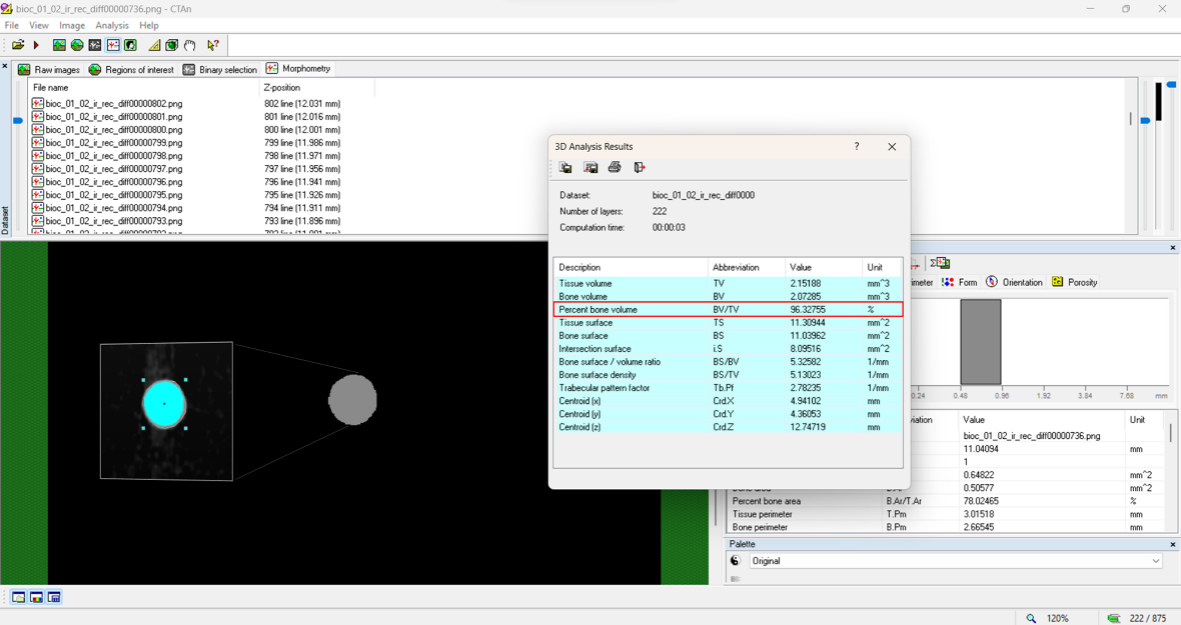
Representative image of the quantitative assessment of the percentage of filling material using the CTAn software

 The artificial canals were scanned using micro-CT (SkyScan 1176; Bruker, Kontich, Belgium) after obturation using defined parameters after the pilot test: isotropic voxel of 8.74 µm, copper and aluminum filter, exposure time of 1900 ms, rotation step 0.5, rotation angle 180°, frame 4, 80 kV and 310 uA. The images obtained were reconstructed using NRecon software (NRecon v.1.6.3, Bruker) and quantitatively analyzed by CTAn software (CTAn v1.15.4.0, Bruker). The percentage of the volume of the filling material (sealer and gutta-percha) and the percentage of voids were quantified for the curved artificial main canal and the simulated lateral canals in the cervical, middle, and apical thirds. The volume of interest (VOI) was selected in all extensions of the main canal and for each of the lateral canals. An interpolated region of interest was defined to exclude the acrylic and artifacts. After that, the grayscale range needed to recognize each object of study was determined with a density histogram by using adaptive thresholding. The threshold level for both materials in the simulated canals was 90-255. To obtain the percentage of the volume of the filling material, “the percentage bone volume (BV/TV)” shown in the 3D analysis in the software CTAn was considered (Figure 2), and the percentage of voids was determined using the following formula: [Percentage of voids = 100 - percentage of the volume of the filling material]. Three-dimensional models were created by CTVox software (v.3.2, Bruker). It is important to highlight that a single operator previously trained and calibrated executed all analysis

### Flow test following ISO 6876/2012 standards and additional analysis

Flow test was performed based on ISO 6876 standards ^5^. After manipulation, 0.05 ± 0.005 ml of each material was placed in the center of a glass plate using a graduate syringe (n=10). At 180±5 seconds after the initial manipulation, another glass plate (20 g) and a metal weight (100g) were placed over the sealer and kept for 10 minutes. After that, the maximums, and minimums diameters of the materials on the glass plate were measured by a digital caliper (Mitutoyo, Suzano, São Paulo, Brazil). When a difference of less than 1 mm between the diameters was observed, the mean value was recorded. The second analysis was performed by photographing the set (glass plate and sealer) next to a millimeter ruler. The images obtained were evaluated using ImageJ software (National Institutes of Health, Bethesda, USA), to obtain the flow area of the material expressed in mm^2^ as proposed by Tanomaru-Filho et al. ^6^.

**Figure 3:**
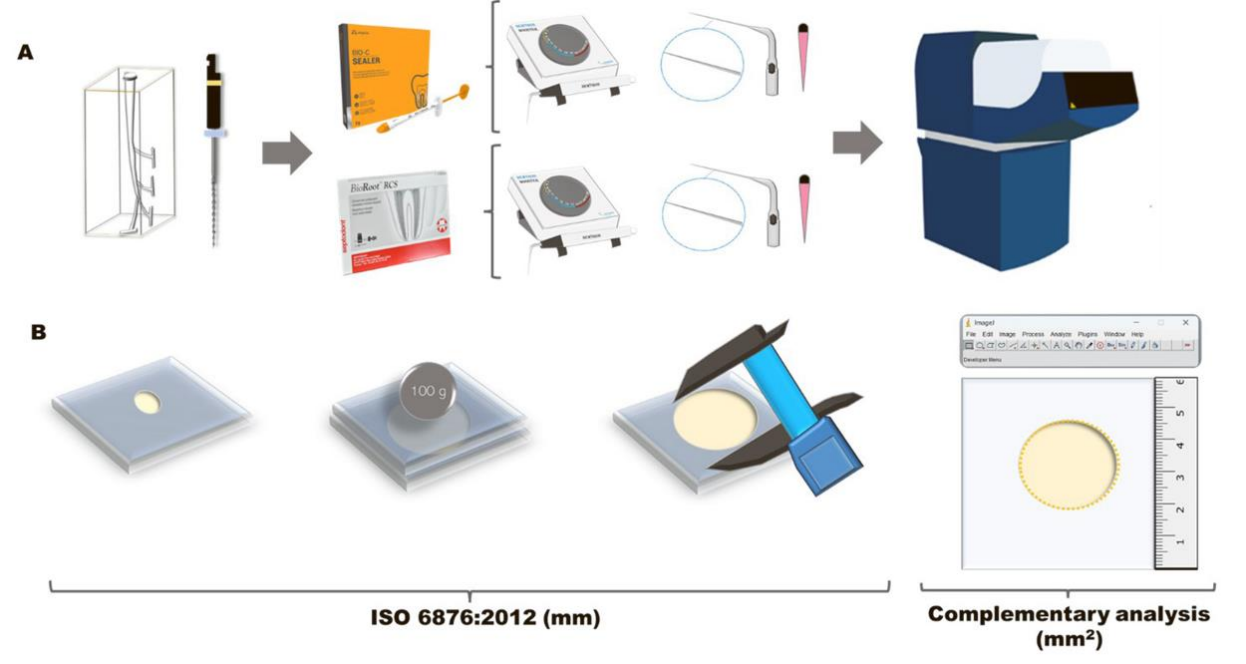
Schematic figure representing the methodology. (A) Preparation and obturation of the simulated curved canals using single-cone technique and Bio-C Sealer or BioRoot RCS without or with ultrasonic agitation and scanning with micro-CT - 8.74 µm to evaluate the percentage of voids. (B) Flow assessment according to ISO 6876:2012 (mm) and complementary analysis (mm^2^).

A schematic methodological demonstrating the filling capacity and flow can be seen in Figure 3.

### Statistical analysis

All data were analyzed using GraphPad Prism 7.00 statistical software (GraphPad Software, La Jolla, CA, USA). The normal distribution of data was confirmed by the Shapiro-Wilk test. Comparisons between groups were performed using ANOVA and Tukey tests. The significance level was 5% for all analyses.

## Results

### Filling Capacity

**Figure 4:**
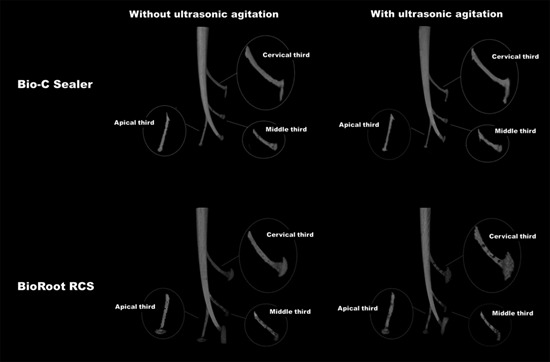
Three-dimensional reconstructions of micro-CT showing the filling of the simulated curved canals after obturation with Bio-C Sealer or BioRoot RCS without or with ultrasonic agitation.

Bio-C Sealer and BioRoot RCS showed a similar percentage of filling material in the curved main canal independent to use the ultrasonic agitation (p>0.05). BioRoot RCS with agitation presented a higher percentage of voids in the lateral canals compared to Bio-C Sealer without or with agitation (p<0.05). Ultrasonic agitation of BioRoot RCS resulted in a higher percentage of voids compared to BioRoot RCS without agitation in the lateral canal of the apical third (p<0.05). Ultrasonic agitation did not influence the filling ability of Bio-C Sealer in the lateral canals (p>0.05) (Table 1, Figure 4).

**Table 1:** Mean and standard deviation of the percentage of filling material and voids by Bio-C Sealer or BioRoot RCS without and with ultrasonic agitation in simulated curved canals

		Bio-C Sealer without agitation	Bio-C Sealer with agitation	BioRoot RCS without agitation	BioRoot RCS with agitation
Filling material (%)	Principal canal	99.82 ± 0.22^a^	99.49 ± 0.59^a^	99.66 ± 0.14^a^	99.30 ± 0.58^a^
	Cervical third	96.97 ± 3.13^a^	98.11 ± 1.06^a^	85.46 ± 4.68^ab^	77.16 ± 8.76^b^
	Middle third	91.99 ± 5.53^a^	96.20 ± 2.21^a^	73.43 ± 6.05^b^	73.48 ± 5.90^b^
	Apical third	96.15 ± 3.99^a^	96.96 ± 3.74^a^	94.50 ± 3.12^a^	83.52 ± 5.01^b^
Voids (%)	Principal canal	0.18 ± 0.22^a^	0.57 ± 0.64^a^	0.33 ± 0.14^a^	0.31 ± 0.58^a^
	Cervical third	3.02 ± 3.13^a^	1.78 ± 1.06^a^	14.53 ± 4.68^ab^	22.83 ± 8.76^b^
	Middle third	8.01 ± 5.53^a^	4.15 ± 2.26^a^	25.45 ± 6.15^b^	26.51 ± 5.92^b^
	Apical third	3.84 ± 3.99^a^	3.61 ± 2.74^a^	5.49 ± 3.12^a^	16.47 ± 5.04^b^

Different superscript lowercase letters in the same line indicate a statistical difference between the groups (p<0.05).

### Flow

The results of the flow test using ISO 6876 and additional analysis are described in Table 2. Bio-C Sealer showed greater flow in both analyses (mm e mm^2^) compared to BioRoot RCS (p<0.05).

**Table 2:** Mean and standard deviation of the flow in mm and mm^2^ of the Bio-C Sealer or BioRoot RCS

	Bio-C Sealer	BioRoot RCS
Flow (mm)	31.16 ± 1.30^a^	19.16 ± 0.72^b^
Flow (mm^2^)	860.2 ± 35.22^a^	287.0 ± 26.89^b^

Different superscript lowercase in the same line indicates a statistical difference between the groups (p<0.05).

## Discussion

 This study evaluated the effect of ultrasonic agitation on the filling ability of ready-to-use Bio-C Sealer or powder-liquid BioRoot RCS. Based on the current findings, significant differences were detected in the percentage of filling material and voids between the sealers evaluated, leading to the rejection of the first null hypothesis.

 The results of the present study revealed lower filling when using ultrasonic agitation of BioRoot RCS in the simulated lateral canals concerning Bio-C Sealer, regardless of the agitation protocol. Lower flow ^22^, a higher percentage of voids ^15^, and pores ^23^ were reported for BioRoot RCS when compared to AH Plus sealer. On the other hand, Bio-C Sealer demonstrates greater flow than AH Plus and ready-to-use calcium silicate TotalFill BC Sealer (FKG Dentaire SA, La Chaux-de-Fonds, Switzerland) ^9^. Furthermore, adequate results on the filling capacity of flattened root canals were observed for Bio-C Sealer (2, 3). Thus, we can suggest that excellent flow associated with filling capacity can explain the lower percentage of voids observed for Bio-C Sealer in the lateral canals of this study.

 Interestingly, our results demonstrated a higher percentage of voids when BioRoot RCS was agitated compared to non-agitated in the lateral canals of the apical third. According to this finding, ultrasonic agitation of endodontic sealer was not related to greater filling in extracted human teeth ^17^. Furthermore, physical changes have been described for Bio-Root RCS after heat application, such as reduced setting time and lower flow ^24^. Thus, the increase in the temperature caused by ultrasonic agitation may have negatively influenced the filling capacity of Bio-Root RCS in the lateral canals of the apical third of this study. In addition, we can speculate that the heat resulting from ultrasonic vibration may have affected the setting time and flow of this material since a previous study reported adequate physicochemical properties for bioceramic materials in powder-liquid presentation ^7^.

Adequate filling capacity (near to 100%) was observed in the simulated curved main canals of this study for both materials regardless of the ultrasonic agitation. This result may be related to the properties of sealers based on calcium silicate (7, 0), as well as the use of simulated circular canals in acrylic resin blocks ^21^ ^,^ ^25^. It is important to highlight that the use of simulated canals may not completely represent a clinical application ^14^ ^,^ ^21^ ^,^ ^25^, resulting in a limitation of the present investigation. Therefore, future research should focus on the use of extracted human teeth with root canals presenting anatomical complexities to further explore the effects of ultrasonic agitation on the filling capacity of bioceramic sealers. The results of the present investigation can be used as a starting point for future comparisons.

 In the present study, sealer flow was evaluated following the ISO 6876/2012 guidelines (linear measurement expressed in mm) ^5^ and through complementary analysis considering the material flow area (mm^2^) ^6^. The additional flow analysis in mm^2^ was used to complement the conventional ISO standard, considering that it does not evaluate the whole area occupied by endodontic sealers ^6^. Therefore, the flow results in mm^2^ from this study can provide a better understanding of the flow capacity of bioceramic sealers in canals with anatomical complexities. Our results revealed that both sealers accomplish the ISO 6876 standards (≥ 17 mm), as previously reported in studies ^9^ ^,^ ^10^ ^,^ ^23^. However, higher flow values were observed in Bio-C Sealer compared to BioRoot RCS in both analyses (mm and mm^2^), leading to the rejection of our second null hypothesis. High values were also reported for Bio-C Sealer in both flow analyses, being higher than AH Plus and TotalFill BC Sealer ^9^. These results may be correlated with the findings of the present study regarding the adequate filling capacity for Bio-C Sealer after obturation of simulated curved and lateral canals regardless of the use of ultrasonic agitation. 

The present investigation used different methodological approaches to allow an integrative analysis of the results of the filling and flow capacity of bioceramic endodontic sealers in areas of anatomical complexity, such as curvatures and lateral canals, which represent a greater difficulty for adequate preparation and filling ^1^. Therefore, the current findings can provide greater support for the clinician before indicating or not the use of the ultrasonic agitation protocol for bioceramic sealers in powder-liquid or ready-to-use form, especially in cases of complex root anatomies.

 Within the limitations of this *in-vitro* study, it can be concluded that Bio-C Sealer and BioRoot RCS present adequate filling capacity in the simulated curved principal canals regardless of the use of ultrasonic agitation. However, BioRoot RSC showed more voids when agitated in the lateral canal of the apical third. Although both sealers present flow following ISO 6876 standards, Bio-C Sealer demonstrates higher values than BioRoot RCS in both analyses (mm and mm^2^).
